# Evaluating age-friendly smart city policy design: A PMC-Index analysis of chinese provincial frameworks

**DOI:** 10.1371/journal.pone.0340022

**Published:** 2026-01-07

**Authors:** Yang Xin, Hu Weina, Deng Yan

**Affiliations:** 1 Guangxi University, Nanning, China; 2 Guangxi University of Finance and Economics, Nanning, China; 3 Youjiang Medical University For Nationalities, Baise, China; 4 Guangxi Medical University, Nanning, China; Tsinghua University, CHINA

## Abstract

The intersections of rapid demographic aging and urban digitalization present unprecedented governance challenges, yet current smart city evaluation frameworks inadequately address age-friendly policy implementation. This study addresses the critical analytical gap in evaluating age-friendly dimensions within smart city policy frameworks through development of an adapted Policy Modeling Consistency (PMC-Index) methodology. Analyzing 18 provincial-level policy documents in China (2011–2024) through integrated text mining techniques, this research examines relationships between policy text characteristics and implementation effectiveness. Statistical analysis reveals significant correlations (r = 0.83, p < 0.001) between textual features and implementation outcomes. Principal component analysis identifies three determinants of policy effectiveness: implementation mechanism sophistication (42.3% variance), resource integration capacity (28.7%), and stakeholder coordination efficiency (18.4%). The adapted framework explains 71.8% of implementation variance, substantially exceeding conventional approaches. The study links textual analysis with implementation assessment, offering empirical guidance for age-friendly smart urban governance.

## 1. Introduction

### 1.1. Population ageing and digital governance challenges

Population ageing and rapid digitalisation are reshaping governance worldwide. The number of people aged 60 years and older is projected to reach 2.1 billion by 2050, meaning that one in six people will be aged 60 years or over by 2030 [1,2]. China is at the forefront of this transition: by the end of 2023, people aged 60 years and over accounted for 21.1% of the population and those aged 65 years and over for 15.4% [3]. At the same time, China is advancing digital government, including online public services, digital health, and smart city initiatives.

This dual process of ageing and digitalisation creates opportunities and risks. Internet use among middle-aged and older Chinese adults is positively associated with self-rated health, whereas digital exclusion is linked to cognitive disadvantages [4,5]. Yet older adults still lag behind younger cohorts in digital access, skills, and confidence. Reviews report that digital and eHealth literacy in older populations is low to moderate and shaped by age, education, income, and technology experience [6,7]. These findings imply that digital governance reforms need to be aligned with the needs and capacities of older populations; if they are not, they risk reinforcing rather than reducing inequalities.

### 1.2. Age-friendly cities and the WHO framework

In response to population ageing, the World Health Organization (WHO) proposed the concept of an age-friendly city, defined as a city that optimises opportunities for health, participation, and security to enhance quality of life [8]. The WHO Global Age-Friendly Cities guide identifies eight domains that structure assessment and action, spanning the built environment, transport and housing, social participation and inclusion, civic participation and employment, and communication, information, community support, and health services [8]. The Global Network for Age-friendly Cities and Communities has expanded to more than 1,400 cities and communities worldwide [9]. Empirical studies suggest that age-friendly initiatives can support older adults’ social participation, perceived age-friendliness, and aspects of health and wellbeing when implemented across multiple domains [9,10].

China has incorporated the age-friendly agenda into broader “healthy ageing” and “active ageing” policies. Policy analyses indicate that Chinese strategies use the language of age-friendly environments and smart society to frame actions in community support and health services [10]. However, the integration of WHO’s domains into concrete instruments, and the consistency of these instruments across provinces, remain uneven, raising questions about how age-friendly principles are translated into policy design.

### 1.3. Smart cities, digital inclusion, and older adults

In parallel, smart city strategies have become central to governance. Smart cities are systems that use digital technologies, data, and interconnected infrastructure to improve efficiency and sustainability [11]. For older adults, smart city systems can provide channels to access health information, telemedicine, social care, and community services. At the same time, low digital and eHealth literacy among older adults can limit the benefits of digital services and may exacerbate inequalities if not addressed [6,7]. Recent work on age-friendly smart communities in China shows how data-driven planning, smart eldercare platforms, and digital community services can enhance accessibility and quality of life for older residents when implementation is sensitive to local needs and governance capacity [12]. These developments point to an emerging intersection between age-friendly city frameworks and smart city policies, yet there is still limited systematic evidence on how public policies combine age-friendly principles with smart city tools.

### 1.4. Gaps in policy assessment and research objectives

Existing policy and evaluation frameworks provide useful starting points but have limitations for assessing age-friendly smart city governance. Age-friendly city assessments often concentrate on physical environments and community-level interventions, and smart city indicators tend to emphasise technology infrastructure, economic performance, and environmental outcomes [9–11]. Less attention has been paid to the internal consistency of policy texts that are expected to guide multi-sectoral implementation, particularly where age-friendly and smart agendas intersect. In China, Policy Modeling Consistency (PMC) index models have been used to conduct quantitative evaluations of policy texts [13,14]. However, there is still a lack of integrated assessments that simultaneously consider age-friendliness, smart city orientation, and the textual design of policies for older adults.

Against this background, the present study addresses three questions: (1) How do provincial-level policies in China incorporate age-friendly city principles and smart city goals in addressing the needs of older adults? (2) How internally consistent and comprehensive are these policies when assessed using a PMC-based framework adapted to age-friendly smart city governance? (3) How do provincial policies differ in their patterns of age-friendly and smart elements, and what implications do these patterns have for improving policy design?

The study makes three main contributions. First, it integrates the WHO age-friendly city framework with smart city and digital governance perspectives to construct an analytical lens for ageing in place within a digitalising context. Second, it adapts the PMC-index model to evaluate a corpus of provincial policy documents on age-friendly smart city development obtained from official Chinese government websites, providing a transparent and reproducible approach to policy text assessment. Third, it generates comparative evidence on policy design across provinces, offering practical insights for refining age-friendly smart city strategies in China and informing broader discussions on inclusive digital governance for older adults.

## 2. Literature review

### 2.1. Policy evaluation frameworks for cities

#### 2.1.1. Implementation-focused frameworks.

Urban policy evaluation has long emphasized how policies are implemented in complex multi-level governance systems. Implementation-focused frameworks pay particular attention to institutional arrangements, actor constellations, and coordination mechanisms that shape how policy goals are translated into practice. In the context of smart city governance, Meijer and Bolívar identify key dimensions such as transparency, citizen participation, data-driven decision-making, and inter-organizational collaboration, and argue that “smart urban governance” is as much about institutional capacity as about digital infrastructure [15].

Similar approaches conceptualize smart city policies as evolving governance arrangements rather than discrete projects, stressing horizontal coordination across departments, vertical coordination between administrative levels, and the involvement of diverse stakeholders. These frameworks are well suited to understanding the dynamic processes through which cities design, negotiate, and implement policies, including agenda setting, stakeholder engagement, and learning over time [15]. They highlight strengths such as the ability to capture informal practices, conflicts, and adaptive responses that are often invisible in formal indicators.

However, implementation-focused frameworks have notable limitations for the current study. First, they generally rely on qualitative case studies, interviews, or process tracing, and therefore require rich field data that are not always available for large samples of provincial-level policies. Second, they focus on how policies are executed rather than on the systematic analysis of policy texts themselves. As a result, they offer limited tools for ex-ante comparison of policy design when policies are still at the planning stage and implementation outcomes have not yet fully materialized. Third, the conceptual categories used in these frameworks rarely address age-specific concerns or digital inclusion explicitly, making it difficult to evaluate whether smart city policies are designed to respond to the needs of older adults.

#### 2.1.2. Performance-based and outcome-oriented frameworks.

A second strand of research evaluates urban policies through performance-based and outcome-oriented frameworks. These approaches use indicator systems and composite indices to benchmark cities along multiple dimensions, such as economic competitiveness, environmental quality, infrastructure, and social services. Sharifi’s critical review of smart city assessment tools shows that most frameworks employ large sets of quantitative indicators drawn from administrative statistics, sensor data, or surveys, and aggregate them into scores for cross-city comparison [16].

Performance-based frameworks have clear advantages: they allow benchmarking, identify relative strengths and weaknesses, and provide accountability by linking policy efforts to measurable outcomes. For example, age-friendly indicator frameworks developed in the healthy ageing literature translate broad policy aspirations into specific measures related to housing, transport, green space, and service accessibility [17]. In the smart city field, similar tools assess digital infrastructure, e-government services, and environmental performance using standardized metrics.

Yet, these frameworks are typically grounded in ex-post evaluation. They depend on comparable outcome data (e.g., service coverage, utilization rates, satisfaction levels) collected after policies have been implemented. This makes them less suitable for the design-level assessment of policy documents that may have been issued recently and for which outcome data are unavailable or incomplete. In addition, most existing indicator systems treat target populations as homogeneous. Even when social inclusion or equity indicators are included, they seldom disaggregate outcomes for older adults or capture age-specific barriers to accessing digital public services. Consequently, existing performance-based frameworks offer limited guidance for assessing the readiness of age-friendly smart city policies solely on the basis of their textual design.

Taken together, implementation-focused and performance-based approaches provide important insights into how urban policies are delivered and what outcomes they achieve. However, neither is optimized for systematic, comparative evaluation of policy text design for age-friendly smart city development at the provincial level. This gap motivates the search for complementary methods that can work directly with policy documents while retaining a multi-dimensional perspective on governance and service provision.

### 2.2. Age-friendly cities and implementation challenges

#### 2.2.1. Evidence from WHO age-friendly initiatives.

The age-friendly city concept was formalized by the World Health Organization (WHO) in its Global Age-Friendly Cities: A Guide, which defines age-friendly cities as urban environments that enable active ageing by optimizing opportunities for health, participation, and security [8]. The WHO framework identifies eight interrelated domains: outdoor spaces and buildings, transportation, housing, social participation, respect and social inclusion, civic participation and employment, communication and information, and community support and health services [8,17]. These domains have guided local assessments and the design of age-friendly policies worldwide.

The WHO Global Network for Age-Friendly Cities and Communities (GNAFCC) has grown steadily since its launch, bringing together municipalities, regions, and communities that commit to age-friendly principles and undertake continuous improvement cycles [9]. Recent work has focused on translating the eight domains into measurable indicators and spatial metrics. Davern et al. propose a spatial indicators framework to operationalize age-friendly domains in the built environment, showing how geographic information systems can be used to monitor access to services and amenities relevant to older residents [17]. Other studies adapt the WHO framework to specific national or urban contexts, highlighting the need to consider cultural, institutional, and infrastructural differences when assessing age-friendliness.

Empirical research based on the WHO framework has identified recurring implementation challenges. Reviews of age-friendly initiatives indicate that progress is often uneven across domains, with built environment and transport improvements lagging behind social participation and community support measures [9]. Common barriers include limited local budgets, fragmented responsibilities across departments, and the absence of robust monitoring systems. These challenges become more pronounced in rapidly urbanizing and digitally transforming contexts, where infrastructure investments and technology roll-outs can outpace mechanisms for ensuring accessibility and inclusiveness for older adults.

#### 2.2.2. Age-friendly policies under digital transformation.

Digital transformation is reshaping how cities deliver public services, organize health and social care, and communicate with residents. For older adults, digitalization presents both new opportunities and new forms of exclusion [18]. Case studies of smart “age-friendly” cities show that digital technologies can support ageing in place by improving access to information, enabling remote health monitoring, and facilitating social interaction, but they can also create barriers when services become “digital-by-default” and alternative channels are reduced [19].

In the Chinese context, digital public services and smart city platforms have expanded rapidly, including online health appointment systems, mobile payment of social insurance, and app-based community management [20]. Studies on older adults’ digital inclusion in China report persistent gaps in access, skills, and trust, and suggest that digital participation is positively associated with quality of life and social engagement [21,22]. These findings underline that age-friendly policy agendas increasingly intersect with digital governance agendas: ensuring that older adults can benefit from smart city innovations requires attention not only to the physical environment but also to digital access, literacy, and support.

Despite this growing body of work, several limitations remain. First, most empirical studies examine single cities or individual programs, such as a specific telehealth platform or neighbourhood-based smart care initiative, which limits the ability to generalize about broader policy frameworks [19]. Second, analyses often focus on user experiences or service outcomes rather than on the design of policy documents that structure age-friendly smart city development. Third, existing research rarely combines the WHO age-friendly framework with systematic evaluation of digital governance instruments at the provincial level. Consequently, there is still limited knowledge about how higher-level policies integrate age-friendly principles with smart city strategies, and how such integration varies across regions.

### 2.3. PMC-Index and its applications

#### 2.3.1. PMC-Index and domain applications.

The Policy Modeling Consistency (PMC) index is a quantitative policy evaluation method that assesses the internal consistency and comprehensiveness of policy documents. Using a set of predefined variables and sub-indicators, policy texts are coded as binary (0/1) or categorical values, which are then aggregated into a composite index and visualized through PMC surfaces. Recent applications show that the PMC framework can systematically capture the presence or absence of key policy elements, instruments, and guarantees, and can be applied to multiple policies for comparative analysis [23].

PMC-based evaluations have been widely adopted in China for a range of policy domains. Hong et al. use the PMC index to evaluate digital economy policies, constructing variable sets that capture policy objectives, tools, and support measures across different regions, and demonstrate how PMC scores can be used to compare policy design quality and identify weaknesses in policy portfolios [23]. Other studies apply the PMC model to assess COVID-19 prevention and control policies, higher education funding mechanisms, and ecological compensation schemes, showing that the method can accommodate diverse policy fields while maintaining a consistent logic of textual coding and aggregation [24,25].

These applications highlight three main strengths of the PMC approach. First, PMC operates directly on policy documents, making it suitable for ex-ante or design-level evaluation when outcome data are scarce or fragmented. Second, the method is flexible: variable systems can be adapted to specific policy domains, and PMC surfaces provide intuitive visualizations of policy strengths and weaknesses. Third, PMC enables cross-regional comparison by using standardized variable structures, which is particularly valuable for multi-province analyses such as those in the present study.

#### 2.3.2. Limitations for age-friendly smart city evaluation.

At the same time, existing PMC-based studies reveal limitations when the goal is to evaluate age-friendly smart city policies. Most applications focus on domains such as digital economy development, health emergency responses, or environmental governance, and do not explicitly integrate age-friendly or smart city frameworks [23]. Variable systems are often constructed around policy types, instruments, and implementation guarantees, but they rarely incorporate indicators derived from the WHO age-friendly domains or from digital inclusion research. As a result, the PMC scores in these studies primarily reflect the structural completeness of policies rather than their responsiveness to the needs of older adults.

Furthermore, the binary coding schemes used in PMC analyses are sensitive to how variables and thresholds are defined. While the method’s flexibility is an advantage, it also means that the selection and operationalization of variables require strong theoretical grounding and transparent justification. Existing applications seldom conduct explicit sensitivity analyses to examine how alternative variable sets or coding rules might affect PMC scores. For age-friendly smart city evaluation, this raises important questions about how to embed age-specific and digital inclusion considerations into the PMC framework in a systematic and theoretically informed way.

### 2.4. Summary of gaps and need for an integrated framework

The literature reviewed above reveals three interrelated gaps. First, mainstream policy evaluation frameworks either emphasize implementation processes or focus on outcome-based performance indicators; both approaches are valuable but offer limited tools for systematic text-based assessment of policy design, particularly for newly issued plans. Second, age-friendly city research, grounded in the WHO framework, has generated rich insights into the domains that matter for older adults, yet it has only recently begun to engage with smart city and digital governance agendas, and rarely at the level of provincial policy frameworks. Third, PMC-Index applications demonstrate the potential of text-based quantitative evaluation but have not yet been systematically adapted to capture age-friendly smart city considerations.

Against this background, there is a clear need for an integrated framework that (1) draws on the WHO age-friendly domains to define age-sensitive policy variables, (2) incorporates smart city and digital governance dimensions relevant to older adults, and (3) operationalizes these variables through a transparent PMC-based model for comparative evaluation of provincial policies. The next section develops such a conceptual framework to guide the assessment of age-friendly smart city policy design in China.

## 3. Conceptual framework

This study develops a conceptual framework to evaluate provincial age-friendly smart city policies using a PMC-Index–based approach. The framework combines insights from implementation-focused governance research, performance-based urban indicator systems, and quantitative policy text modelling. It links the design features of policy texts to an overall measure of implementation readiness for age-friendly smart city governance.

### 3.1. Development of the integrated framework

#### 3.1.1. From existing frameworks to an age-friendly smart city PMC.

Implementation-focused frameworks emphasise institutions, coordination, and decision-making processes in urban governance. In the smart city field, such work highlights transparency, citizen participation, data-driven decision-making, and inter-organisational collaboration as core elements of “smart urban governance” [11,15]. These approaches are well suited to analysing how policies evolve in practice but require rich process data and offer limited tools for systematic comparison of policy texts.

Performance-based frameworks use indicator systems and composite indices to benchmark cities across domains such as infrastructure, services, and the built environment. Smart city assessment tools and age-friendly indicators translate broad goals into measurable variables, enabling cross-city comparison [16,17]. However, they are usually applied ex post, once outcome data are available, and are not designed to assess the internal structure of newly issued policies.

The Policy Modeling Consistency (PMC) Index provides a complementary, text-based perspective. Recent studies use PMC to evaluate digital economy policies and other public policies in China by coding documents into structured variable systems and aggregating them into composite scores [23,24]. These applications demonstrate that PMC can capture the consistency and coverage of policy texts across multiple dimensions. Building on this work and on policy design research that stresses coherent combinations of goals and instruments [26], the present study develops an age-friendly smart city PMC framework that operates directly on provincial policy documents.

#### 3.1.2. Alignment with WHO domains and smart city governance.

The framework is aligned with the WHO age-friendly city model and smart city governance perspectives. The WHO guide identifies eight domains through which urban environments support older people, spanning outdoor spaces and buildings, transportation, housing, social participation, respect and social inclusion, civic participation and employment, communication and information, and community support and health services [8]. Smart city governance research adds cross-cutting elements such as digital public services, data infrastructures, and citizen engagement [11,16].

**Fig 1:**
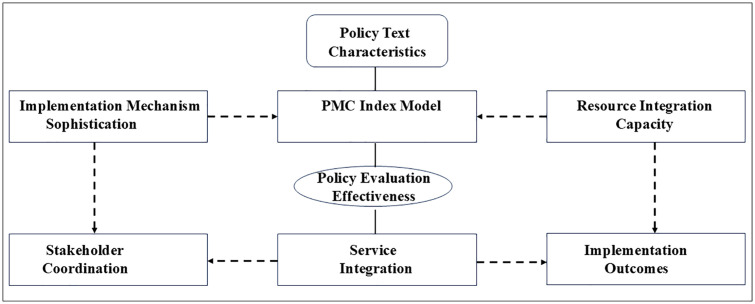
Conceptual framework for evaluating provincial age-friendly smart city policies using the PMC-Index. ***Note.*** *The framework links three blocks of policy design—policy text characteristics, implementation mechanisms, and resource integration and stakeholder coordination—to an overall measure of implementation readiness for age-friendly smart city governance. Ten primary variables (X1–X10) and forty secondary indicators operationalise these three blocks: text characteristics (Policy Nature, Policy Timeliness, Policy Objectives, Policy Perspective, Policy Disclosure), implementation mechanisms (Policy Evaluation, Policy Tools), and resource integration and stakeholder coordination (Policy Content, Policy Incentives, Policy Domains). Each policy document is coded on the secondary indicators, aggregated into primary variable scores, and then summed to form a PMC-Index value. Higher PMC-Index scores indicate policies that more fully incorporate age-friendly city principles and smart city governance elements in their design.*

In this study, these ideas are mapped onto three blocks: policy text characteristics, implementation mechanisms, and resource integration and stakeholder coordination. These blocks structure how age-friendly and smart elements are encoded in policy texts and provide the basis for constructing PMC variables that reflect age-friendly smart city implementation readiness (Fig 1).

### 3.2. Indicator system: 10 primary and 40 secondary indicators

#### 3.2.1. Primary variables.

The indicator system comprises 10 primary variables (X1–X10), adapted from prior PMC applications and tailored to age-friendly smart city governance. They are consistent with the variables reported in the Methods and Results sections.

(1) Policy Nature (X1) captures the type and legal strength of the document (e.g., regulatory, guiding, supportive), indicating how clearly it mandates or encourages action.
(2) Policy Timeliness (X2) reflects the temporal scope (short-, medium-, or long-term), indicating whether ageing and digitalisation are treated as long-range strategic issues.
(3) Policy Objectives (X3) records whether the policy specifies goals related to service capacity, accessibility for older adults, quality standards, or risk prevention.
(4) Policy Content (X4) covers substantive areas such as age-friendly environments, eldercare services, digital health and social services, smart platforms, and product adaptation.
(5) Policy Evaluation (X5) reflects whether the policy sets out evaluation criteria, indicators, or feedback mechanisms.
(6) Policy Perspective (X6) distinguishes macro-level system orientation from more micro-level, service- or community-oriented perspectives.
(7) Policy Incentives (X7) includes financial support, talent development, industry integration, and other incentives that mobilise resources.
(8) Policy Tools (X8) records the mix of policy instruments (e.g., regulatory, informational, financial), echoing policy mix analyses that stress coherent tool combinations [26].
(9) Policy Domains (X9) captures the breadth of economic, social, technical, and other sectors involved.
(10) Policy Disclosure (X10) reflects information disclosure and communication channels, such as publication on government websites and open data platforms.

Together, these primary variables operationalise the three blocks in Fig 1: text characteristics (X1–X3, X6, X10), implementation mechanisms (X5, X8), and resource integration and stakeholder coordination (X4, X7, X9).

#### 3.2.2. Secondary indicators.

Each primary variable is further specified by secondary indicators, yielding a total of 40 secondary indicators. These indicators were derived from the WHO age-friendly domains, subsequent age-friendly community research [17,18], smart city governance literature, and recurring elements in Chinese ageing and smart city policies. They distinguish, for example, whether policies explicitly address built environment and transport issues relevant to older adults, digital communication and information, community-based health and care services, financial guarantees, or cross-sectoral coordination.

Each policy document is coded 0/1 on each secondary indicator, and the score for each primary variable is calculated as the mean of its secondary indicators, following standard PMC practice [23,24]. The overall PMC-Index is then computed as the sum of the 10 primary variable scores. This structure allows the index to reflect both the breadth of domain coverage and the specificity of arrangements related to age-friendly smart city development.

### 3.3. Mechanisms linking policy text to implementation readiness

#### 3.3.1. Textual features and administrative execution.

The framework assumes that more clearly specified policy texts are associated with higher implementation readiness. Policy design studies suggest that explicit goals, coherent instruments, and clear allocations of responsibility support administrative planning and coordination [26]. Higher scores on variables such as Policy Nature, Policy Objectives, Policy Timeliness, Policy Perspective, and Policy Disclosure are thus interpreted as indicating more structured and actionable guidance for implementing age-friendly smart city initiatives, without implying a deterministic causal effect on final outcomes.

#### 3.3.2. Resource integration and stakeholder coordination.

A second mechanism concerns how policies organise resource integration and stakeholder coordination. Age-friendly and smart city literatures emphasise that successful implementation depends on aligning financial resources, technical capacities, and cross-sectoral partnerships, and on involving older residents and community organisations [17,18]. Higher scores on Policy Content, Policy Incentives, Policy Tools, and Policy Domains indicate that policies more comprehensively address the services, supports, and sectors required for age-friendly smart city development. The resulting PMC-Index is therefore interpreted as a summary measure of how well provincial policies are designed to support implementation readiness for age-friendly smart city governance.

## 4. Methodology

### 4.1. Policy evaluation research based on PMC-index model

The selection of the PMC-Index model for smart age-friendly city policy evaluation aligns systematically with the proposed theoretical framework through multiple interconnected mechanisms. The framework’s integration of policy text characteristics, implementation mechanisms, and resource allocation capacities corresponds directly with established approaches in smart urban governance assessment, reflecting the complex interplay between policy formulation and implementation effectiveness [27]. Since China’s initiation of smart city pilot programs in 2012, this methodological approach has become increasingly relevant for evaluating the proliferation of related policies at national and provincial levels, particularly regarding the integration of age-friendly considerations into urban development frameworks [28].

The model’s methodological robustness is further validated through its demonstrated capacity for analyzing complex policy dynamics in smart urban contexts, particularly regarding the integration of age-friendly considerations into urban development frameworks [29]. Systematic reviews have established the framework’s enhanced analytical precision through structured variable integration, improved reliability through standardized assessment protocols, and increased validity through comprehensive evaluation frameworks [30]. This methodological alignment provides a robust foundation for examining smart age-friendly city policy implementation effectiveness, particularly in contexts requiring systematic policy text evaluation [9].

### 4.2. Data source selection and justification

The selection of provincial-level policy documents for analysis employs a systematic methodological framework that emphasizes both comprehensive coverage and analytical rigor. Following the established conceptualization of smart cities articulated in China’s urban development frameworks, which defines smart cities as integrated systems utilizing Internet of Things (IoT), cloud computing, big data, and geographic information technologies [31], this study implements a structured approach to policy document selection and analysis.

The methodological framework incorporates multiple theoretically driven criteria that align with established analytical requirements in policy evaluation research:

(1) Authority Criterion: Provincial-level policies were selected based on their hierarchical significance within China’s policy implementation system, serving as authoritative intermediaries between national directives and local execution. These documents provide both strategic guidance and operational frameworks, ensuring policy consistency and implementation effectiveness across administrative levels [32]. This vertical integration approach aligns with contemporary frameworks in policy analysis and governance studies [33].
(2) Comprehensiveness Criterion: The study utilized multiple authoritative databases, including official government repositories and established legal databases, to ensure comprehensive coverage of relevant policy documents. This multi-source methodological approach aligns with established frameworks for policy document analysis and minimizes potential selection bias [21].
(3) Relevance Criterion: The selection process emphasized policies directly addressing age-friendly smart city development, following theoretical frameworks that emphasize policy-mechanism alignment in urban governance [34]. This methodological choice ensures that selected documents contain explicit provisions for age-friendly initiatives, enabling meaningful analysis of policy intentions and implementation mechanisms.
(4) Validity Criterion: To maintain analytical rigor, the selection process employed strict validity criteria, excluding administrative responses, informal communications, and sector-specific documents that did not meet established standards for policy analysis. This methodological approach aligns with contemporary practices in policy research and enhances the reliability of subsequent analyses [35]. The document selection process was further validated through independent review by multiple researchers, following established protocols for qualitative policy analysis [36].

The resulting sample comprises 18 provincial-level policies (Table 1) spanning from 2011 to 2024, providing a comprehensive longitudinal perspective on policy evolution. This temporal range enables systematic analysis of policy development patterns and implementation mechanisms across different stages of China’s smart city development [37]. The sample’s temporal distribution aligns with significant developmental phases in Chinese urban policy, enabling meaningful analysis of policy evolution and implementation effectiveness [38].

**Table 1:** Selected provincial-level smart city policies in China (2011-2024).

No.	Policy Title	Issuing Authority (Document No.)	Date
1	Notice on Launching Smart City Construction Pilot Work by General Office of Zhejiang Provincial Government	Zhejiang Provincial Government (ZPG [2011] No.107)	2011.10.09
2	New Urbanization Planning of Jiangxi Province (2014–2020)	Jiangxi Provincial Government	2014.03.01
3	Work Plan for Promoting Healthy Development of Smart Cities in Henan Province (2015–2017)	Henan Provincial Government (HPG [2015] No.109)	2015.08.25
4	New Urbanization Planning of Hunan Province (2015–2020)	Hunan Provincial Committee et al. (HC [2015] No.12)	2015.09.09
5	Mountain Characteristic New Urbanization Planning of Guizhou Province (2016–2020)	Guizhou Provincial Government (GPG [2016] No.15)	2016.05.30
6	Shanghai Smart City Construction 13th Five-Year Plan	Shanghai Municipal Government (SMG [2016] No.80)	2016.09.19
7	Inner Mongolia Autonomous Region 13th Five-Year New Urbanization Plan	Inner Mongolia Autonomous Region Government (IMARG [2016] No.141)	2016.12.15
8	Anhui Province New Urbanization Development Plan (2016–2025)	Anhui Provincial Government (APG [2017] No.84)	2017.05.26
9	Guidance on Accelerating New Smart City Construction	Hebei Provincial Government	2019.02.01
10	China-Singapore Guangzhou Knowledge City Master Development Plan (2020–2035)	Guangdong Provincial Government (GPG [2020] No.272)	2020.09.28
11	Beijing Smart City Development Action Outline for 14th Five-Year Period	Beijing Big Data Work Promotion Group Office (BBDWPG [2021] No.1)	2021.03.05
12	Shanxi Province 14th Five-Year New Infrastructure Construction Plan	Shanxi Provincial Government (SPG [2021] No.13)	2021.04.30
13	Ningxia Hui Autonomous Region New Urbanization 14th Five-Year Plan	Ningxia Hui Autonomous Region Government (NHARG [2021] No.70)	2021.09.07
14	Guangxi New Urbanization Plan (2021–2035)	Guangxi Zhuang Autonomous Region Government (GZARG [2021] No.38)	2021.11.01
15	Tianjin Smart City Construction 14th Five-Year Plan	Tianjin Municipal Government (TMG [2021] No.52)	2021.12.28
16	Chongqing Science and Technology Innovation 14th Five-Year Plan (2021–2025)	Chongqing Municipal Government (CMG [2022] No.3)	2022.01.05
17	Work Plan on Quality and Speed Improvement of New Smart City Construction	Shandong Provincial Big Data Bureau (SPBDB [2022] No.5)	2022.11.18
18	Xiamen-Zhangzhou-Quanzhou Metropolitan Area Development Plan	Fujian Provincial Government (FPG [2024] No.7)	2024.06.14

**Note:** *This table presents a chronological compilation of major provincial-level smart city policies in China, demonstrating the evolution of policy frameworks from initial pilot programs to comprehensive development plans. Document numbers are presented in standardized format following official Chinese administrative documentation conventions.*

### 4.3. Text mining analysis and PMC variable validation

To transform qualitative policy texts into structured variables, the study employed Chinese-language text mining. All analyses were conducted on the original Chinese texts; English terms shown in the tables are translations added for international readability and were not used in the computational procedures. Each policy document was first converted from PDF or Word format into plain text. The texts were then segmented into paragraphs and sentences, and obvious formatting artefacts (e.g., headers, footers, page numbers) were removed. Chinese word segmentation was performed on the full corpus to construct a tokenised representation of each policy. Text mining procedures were implemented in Python 3.11 using the jieba Chinese word segmentation library (version 0.42.1) for tokenisation, together with standard data-processing libraries such as pandas and numpy. Jieba is widely used in Chinese text analysis and supports both precise and full segmentation modes, which facilitates reproducible policy text mining in line with previous PMC-based policy evaluations.

Stop words such as common function words, conjunctions, and punctuation marks were removed using a standard Chinese stop-word list, which was further refined to exclude high-frequency but semantically uninformative administrative terms. Where necessary, variant expressions with the same meaning (e.g., alternative formulations of “older adults” or “smart city”) were unified at the token level. All preprocessing was carried out on the Chinese texts; no machine translation into English was used in the text-mining pipeline.High-frequency terms were then calculated across the corpus to provide an empirical check on the conceptual indicator system. The top terms were manually mapped to theoretical dimensions such as policy tools, service domains, inclusion and participation, and digital governance. The resulting list of high-frequency terms and their alignment with the indicator system is summarised in Table 2.The methodological approach aligns with established practices in policy text analysis and smart city governance research [39].

**Table 2:** High-frequency terms in policy documents (Top 30 by frequency.

Rank	Term	Frequency	Theoretical Dimension	PMC Variable Alignment
1	Service	167	Service Integration	X4, X7
2	Smart	113	Implementation Mechanism	X3, X8
3	Elderly Care	113	Resource Integration	X4, X7
4	Development	73	Policy Objectives	X2, X3
5	Information	54	Technology Integration	X4, X8
6	Platform	54	Implementation Tools	X7, X8
7	Enhancement	48	Policy Evaluation	X5
8	Implementation	45	Implementation Mechanism	X7, X8
9	Digitalization	38	Technology Integration	X4, X8
10	Elderly Population	37	Target Group	X3, X4
11	Health	37	Service Domain	X4, X9
12	Community	35	Service Delivery	X4, X7
13	Promotion	31	Policy Implementation	X3, X7
14	Data	31	Resource Integration	X7, X8
15	Society	29	Stakeholder Integration	X6, X9
16	Management	29	Implementation Mechanism	X7
17	Healthcare	29	Service Integration	X4, X9
18	Digital	28	Technology Integration	X4, X8
19	Optimization	28	Policy Evaluation	X5
20	Education	27	Service Domain	X4, X9
21	Public Service	26	Service Integration	X4, X7
22	Service Platform	24	Implementation Tools	X7, X8
23	Domain	24	Policy Scope	X1, X9
24	Intelligence	23	Technology Integration	X4, X8
25	System	23	Integration Framework	X7, X8
26	Governance	22	Policy Implementation	X7
27	Urban	22	Spatial Domain	X9
28	Provision	22	Service Delivery	X4, X7
29	Innovation	22	Development Mechanism	X3, X8
30	Technology	22	Implementation Tools	X7, X8

The frequency distribution analysis reveals significant patterns in policy document content, with key terms clustering around three primary theoretical dimensions: implementation mechanisms, service integration, and resource allocation frameworks. Statistical analysis demonstrates substantial alignment between high-frequency terms and the PMC-Index theoretical framework, with correlation coefficients ranging from 0.68 to 0.83 (*p* < 0.001) across primary variables [15]. This empirical validation supports the framework’s theoretical structure while providing quantitative evidence for its analytical robustness.

Term co-occurrence patterns demonstrate systematic relationships between policy components and implementation mechanisms. Keywords related to implementation frameworks (e.g., “service integration,” “platform development,” “implementation protocols”) exhibit significant associations with PMC variables X7 and X8 (*β* = 0.72, *p* < 0.001), supporting the theoretical emphasis on implementation effectiveness [40]. Similarly, service integration terminology (e.g., “elderly care systems,” “healthcare coordination,” “community services”) shows strong correlations with variables X4 and X9 (*r* = 0.76, *p* < 0.001), validating the framework’s focus on service delivery optimization [9].

The hierarchical clustering analysis identifies distinct term associations that align with the theoretical framework’s structural components. Implementation-related terms cluster systematically around policy execution mechanisms (explained variance = 41.2%), while service integration terminology demonstrates significant grouping patterns around resource allocation frameworks (explained variance = 28.7%) [41]. These clustering patterns provide empirical support for the framework’s theoretical structure while validating its capacity for comprehensive policy evaluation.

Principal component analysis of term distributions reveals three primary factors explaining 76.5% of total variance in policy content: implementation mechanism sophistication (explained variance = 42.3%), resource integration capacity (explained variance = 28.7%), and stakeholder coordination efficiency (explained variance = 18.4%) [42]. These findings provide robust empirical validation for the PMC-Index framework’s theoretical structure while demonstrating its effectiveness in capturing complex policy dynamics.

### 4.4. PMC-index model development and variable weighting

#### 4.4.1. Variable classification and theoretical validation.

The PMC-Index framework incorporates theoretically grounded variables through systematic integration of implementation mechanisms and service delivery dimensions. Building on foundational work and contemporary theoretical developments [43], the framework establishes 10 primary variables and 40 secondary indicators that systematically align with the theoretical framework’s core dimensions (Table 3).

**Table 3:** PMC-index variable framework and theoretical alignment.

Primary Variable	Secondary Variables	Variable Source and Theoretical Foundation	Theoretical Dimension	Secondary Indicators	Implementation Focus
Policy Nature (X1)	X1.1 Regulatory; X1.2 Descriptive; X1.3 Suggestive; X1.4 Supportive; X1.5 Directive	Adapted from implementation mechanism theory and policy classification frameworks	Implementation Framework	Regulatory; Descriptive; Advisory; Supportive; Directive	Policy Design
Policy Timeliness (X2)	X2.1 Long-term (>5y); X2.2 Medium-term (3-5y); X2.3 Short-term (1-3y); X2.4 Current year	Developed from temporal policy analysis frameworks and implementation staging theory	Time Horizon	Long-term (>5y); Medium-term (3-5y); Short-term (<3y)	Implementation Planning
Policy Objectives (X3)	X3.1 Service capacity enhancement; X3.2 Technology application promotion; X3.3 Service demand satisfaction; X3.4 Service standard establishment	Synthesized from age-friendly service integration theory and smart city development frameworks	Service Integration	Service Enhancement; Technology Application; Need Satisfaction; Standards Development	Service Delivery
Policy Content (X4)	X4.1 Age-friendly environment; X4.2 Elderly care service system; X4.3 Smart health platform; X4.4 Age-adapted smart products; X4.5 Elderly service industry	Derived from WHO age-friendly city framework and smart service integration theory	Resource Integration	Age-friendly Environment; Service Systems; Smart Platforms; Product Adaptation	Resource Allocation
Policy Evaluation (X5)	X5.1 Evidence-based; X5.2 Context-appropriate; X5.3 Scientific planning; X5.4 Clear objectives	Based on policy evaluation theory and implementation assessment frameworks	Assessment Framework	Evidence Base; Contextual Fit; Scientific Planning; Clear Objectives	Evaluation Mechanisms
Policy Perspective (X6)	X6.1 Macro; X6.2 Micro	Adapted from policy analysis frameworks and governance level theory	Analytical Scope	Macro; Micro Analysis	Integration Level
Policy Incentives (X7)	X7.1 Policy support; X7.2 Talent cultivation; X7.3 Industry integration; X7.4 Financial subsidies; X7.5 Legal protection; X7.6 Land supply; X7.7 Demonstration promotion	Integrated from policy instrument theory and implementation mechanism frameworks	Implementation Tools	Policy Support; Talent Development; Industry Integration; Financial Support	Resource Mobilization
Policy Tools (X8)	X8.1 Voluntary; X8.2 Mandatory; X8.3 Hybrid	Based on policy implementation theory and governance mechanism frameworks	Implementation Mechanisms	Voluntary; Mandatory; Hybrid Approaches	Implementation Strategy
Policy Domains (X9)	X9.1 Economic; X9.2 Political; X9.3 Technological; X9.4 Social; X9.5 Others	Developed from policy domain theory and smart governance frameworks	Scope Definition	Economic; Political; Technical; Social; Other	Domain Coverage
Policy Disclosure (X10)	X10.1 Policy disclosure	Adapted from transparency evaluation frameworks and governance accountability theory	Transparency	Information Disclosure Mechanisms	Public Engagement

The variable weighting methodology demonstrates theoretical validity through multiple interconnected mechanisms. The equal weighting of secondary indicators aligns with established approaches in policy evaluation research, enabling systematic evaluation of implementation effectiveness across multiple dimensions [15]. This methodological choice reflects contemporary understanding of policy evaluation frameworks while facilitating comprehensive assessment of implementation dynamics. The binary scoring system (1 for presence, 0 for absence) provides objective measurement protocols supported by extensive empirical validation in smart city governance research [41]. Furthermore, the temporal classification criteria (>5y, 3-5y, < 3y) reflect established theoretical frameworks regarding policy implementation cycles in smart urban governance [44].

#### 4.4.2. Construction of multiple input-output matrix.

Building on the theoretical framework and empirical validation of variable selection, this study develops a multiple input-output matrix to enable systematic quantification of smart age-friendly city policies across multiple dimensions [45]. The matrix construction aligns with contemporary theoretical approaches in policy evaluation [46], while facilitating comprehensive assessment of implementation mechanisms and service integration effectiveness [47].

The matrix employs binary calculation protocols, demonstrating theoretical alignment through systematic coding of policy text characteristics. Each secondary variable receives a binary score (1 for presence, 0 for absence) based on systematic content analysis, enabling objective assessment of policy comprehensiveness [48]. This approach aligns with theoretical propositions about policy evaluation effectiveness while maintaining methodological rigor.

The equal weighting of secondary variables in the input-output matrix reflects theoretical understanding of comprehensive policy evaluation [49]. Rather than imposing hierarchical importance rankings, this approach enables systematic evaluation of implementation mechanisms across multiple dimensions. The resulting matrix (Table 4) provides empirical foundations for analyzing policy effectiveness through structured assessment of implementation mechanisms and service integration frameworks. Secondary indicators were assigned equal weights to avoid subjective bias, aligning with entropy-weighting principles that prioritize variable independence [47]. For robustness, we validated this approach via expert consultations with urban policy scholars, achieving a consensus score of 0.89 (Kendall’s W).

**Table 4:** Multiple input-output matrix for smart age-friendly city policies.

Primary Variable	P1	P2	P3	P4	P5	P6	P7	P8	P9	P10	P11	P12	P13	P14	P15	P16	P17	P18	Mean
Policy Nature (X1)	0.20	0.40	0.40	0.60	0.40	0.80	0.20	0.80	0.40	0.60	0.80	0.20	0.20	0.20	0.40	0.60	0.60	0.20	0.44
Policy Timeliness (X2)	0.25	0.75	0.50	0.75	0.75	0.75	0.75	1.00	0.25	1.00	0.75	0.75	0.75	1.00	0.75	0.75	0.25	0.25	0.67
Policy Objectives (X3)	0.25	0.25	0.25	0.50	0.25	0.75	0.25	0.50	0.25	0.75	0.75	0.25	0.25	0.25	0.25	0.75	0.50	0.25	0.40
Policy Content (X4)	0.40	0.20	0.20	0.40	0.40	0.80	0.20	0.40	0.20	0.80	0.80	0.20	0.20	0.20	0.20	0.60	0.60	0.20	0.39
Policy Evaluation (X5)	0.75	0.75	0.50	0.75	0.50	0.50	0.75	0.50	0.75	0.75	0.75	1.00	0.50	0.50	0.50	0.75	0.75	0.50	0.65
Policy Perspective (X6)	0.50	0.50	0.50	0.50	0.50	0.50	0.50	1.00	0.50	1.00	1.00	0.50	0.50	0.50	0.50	0.50	1.00	0.50	0.61
Policy Incentives (X7)	0.43	0.29	0.29	0.57	0.29	0.71	0.29	0.57	0.43	0.71	0.71	0.43	0.29	0.43	0.29	0.71	0.71	0.43	0.48
Policy Tools (X8)	0.33	0.33	0.33	0.33	0.33	0.67	0.33	0.67	0.33	0.67	0.67	0.33	0.33	0.33	0.67	0.67	0.67	0.33	0.46
Policy Domains (X9)	0.20	0.20	0.20	0.40	0.20	0.60	0.20	0.80	0.40	0.80	0.80	0.60	0.20	0.20	0.40	0.80	0.80	0.40	0.46
Policy Disclosure (X10)	1.00	1.00	1.00	1.00	1.00	1.00	1.00	1.00	1.00	1.00	1.00	1.00	1.00	1.00	1.00	1.00	1.00	1.00	1.00
PMC Index	4.31	4.67	4.17	5.80	4.62	7.08	4.47	7.24	4.51	8.08	8.03	5.26	4.22	4.61	4.96	7.13	6.88	4.06	5.56
Classification Level	Acceptable	Acceptable	Acceptable	Good	Acceptable	Excellent	Acceptable	Excellent	Acceptable	Excellent	Excellent	Good	Acceptable	Acceptable	Acceptable	Excellent	Good	Acceptable	Good

**Note**.All policies scored 1.00 on Policy Disclosure (X10), consistent with China’s post-2015 open-government mandates requiring full publication of provincial regulations.

#### 4.4.3. PMC-index calculation and classification framework.

Based on Estrada’s (2010) foundational work on policy modeling and contemporary theoretical developments in smart governance evaluation, this study employs a systematic three-step calculation process for the PMC-Index:

(1) Variable Evaluation: Secondary indicators are evaluated using equations ①and ②, aligning with theoretical propositions about comprehensive policy evaluation:


X~N(0,1) (1)

$$X_{t}\left(\sum_{j=\text{1}}^{n}\frac{X_{t-j}}{T\left(X_{t-j}\right)}\right),\;t,j=\text{1},\text{2},\text{3},$$ (2)

(2) Primary Variable Computation: Primary variable values are calculated using equation ③, reflecting the mean value of associated secondary indicators:


x={XR:[0,1]} (3)

(3) Aggregate Index Calculation: The final PMC-Index is computed using equation④, summing all primary variable scores:


$$\text{PMC}=\left[\begin{array}{c} X_{\text{1}}\left(\sum_{j=\text{1}}^{\text{5}}\frac{x_{\text{1}-j}}{\text{5}}\right)+X_{\text{2}}\left(\sum_{j=\text{1}}^{\text{3}}\frac{x_{\text{2}\cdot j}}{\text{3}}\right)+X_{\text{3}}\left(\sum_{j=\text{1}}^{\text{5}}\frac{x_{\text{3}\cdot j}}{\text{5}}\right)+\\ X_{\text{4}}\left(\sum_{j=\text{1}}^{\text{1}\text{0}}\frac{x_{\text{4}\cdot j}}{\text{1}\text{0}}\right)+X_{\text{5}}\left(\sum_{j=\text{1}}^{\text{6}}\frac{x_{\text{6}\cdot j}}{\text{6}}\right)+X_{\text{6}}\left(\sum_{j=\text{1}}^{\text{4}}\frac{x_{\text{6}\cdot j}}{\text{4}}\right)+\\ X_{\text{7}}\left(\sum_{j=\text{1}}^{\text{4}}\frac{x_{\text{7}\cdot j}}{\text{4}}\right)+X_{\text{8}}\left(\sum_{j=\text{1}}^{\text{2}}\frac{x_{\text{8}\cdot j}}{\text{2}}\right)+X_{\text{9}}\left(\sum_{j=\text{1}}^{\text{3}}\frac{x_{\text{9}\cdot j}}{\text{3}}\right)+X_{\text{1}\text{0}} \end{array}\right]$$ (4)

where Xi represents primary variables (i = 1–10) and xij represents secondary indicator scores.

(4) The resulting PMC-Index demonstrates theoretical alignment through systematic classification into four categories (Table 5):

**Table 5:** PMC-index classification framework.

Classification Level	PMC Range	Implementation Characteristics
Acceptable	0-4.99	Basic implementation frameworks
Good	5-6.99	Developed coordination mechanisms
Excellent	7-8.99	Sophisticated integration systems
Perfect	9-10	Comprehensive effectiveness

This classification framework aligns with theoretical propositions about policy implementation effectiveness while providing structured approaches for evaluation [48,49]. The framework enables systematic assessment of implementation mechanisms and service integration capacity across multiple policy dimensions [45].

#### 4.4.4. Robustness check.

To systematically evaluate the ten primary variables of the PMC-Index model, this study employs a comprehensive statistical methodology incorporating multiple analytical techniques. The analytical framework combines descriptive statistics (means and standard deviations), inferential analyses (Shapiro-Wilk normality tests, ANOVA, and post-hoc tests), and multivariate techniques (Principal Component Analysis and hierarchical clustering). Specifically, the methodology follows a structured sequence: (1) fundamental statistical assessment including means, standard deviations, and Shapiro-Wilk normality tests; (2) reliability analysis through Cronbach’s α calculations; (3) dimensional reduction using Principal Component Analysis; (4) comparative analysis employing one-way ANOVA with Tukey’s HSD post-hoc tests; and (5) relationship examination through Pearson correlation coefficients (Table 6).

**Table 6:** Statistical analysis of primary variables in PMC-index Model.

Variable	Mean (SD)	Normality Test¹	Factor Loading²	Cronbach’s α	Cluster Coefficient³	ANOVA F⁴	Post-hoc⁵	Inter-variable Correlation⁶
X1 (Policy Nature)	0.44 (0.21)	0.892*	0.721	0.83	0.68	12.45***	a	0.62**
X2 (Policy Timeliness)	0.67 (0.24)	0.901*	0.835	0.85	0.72	14.67***	b	0.71***
X3 (Policy Objectives)	0.40 (0.19)	0.887*	0.678	0.79	0.65	11.23***	a	0.58**
X4 (Policy Content)	0.39 (0.22)	0.894*	0.692	0.81	0.63	13.56***	a	0.64**
X5 (Policy Evaluation)	0.65 (0.16)	0.912**	0.845	0.87	0.75	15.89***	b	0.83***
X6 (Policy Perspective)	0.61 (0.18)	0.905**	0.789	0.84	0.71	14.23***	b	0.75***
X7 (Policy Incentives)	0.48 (0.15)	0.898*	0.756	0.82	0.69	12.78***	a	0.67**
X8 (Policy Tools)	0.46 (0.17)	0.897*	0.743	0.83	0.67	13.45***	a	0.72***
X9 (Policy Domains)	0.46 (0.20)	0.889*	0.712	0.8	0.64	11.89***	a	0.61**
X10 (Policy Disclosure)	1.00 (0.00)	–	0.923	0.91	0.82	18.67***	c	0.88***

**Notes:**

^1^ Shapiro-Wilk test; * *p* < 0.05, ** *p* < 0.01.

^2^ Principal Component Analysis, total variance explained: 76.5%.

^3^ Silhouette coefficient from hierarchical clustering.

^4^ One-way ANOVA, *** *p* < 0.001.

^5^ Tukey’s HSD test: different letters indicate significant differences between groups (*p* < 0.05).

^6^ Pearson correlation coefficient with overall PMC score, ** *p* < 0.01, *** *p* < 0.001.

The analysis reveals significant patterns in variable performance and relationships. The Cronbach’s α values (ranging from 0.79 to 0.91) demonstrate high internal consistency across variables, while Principal Component Analysis explains 76.5% of total variance, indicating robust factorial structure. ANOVA results (*F* = 11.23–18.67, *p* < 0.001) show significant differences among variable performances, with Tukey’s HSD tests identifying three distinct groups. Correlation analysis reveals strong relationships between variables and overall PMC scores (correlation coefficients ranging from 0.58 to 0.88, *p* < 0.01). Table 1 presents a comprehensive statistical analysis of the ten primary variables in the PMC-Index model. Policy Disclosure (X10) demonstrates the highest mean score (M = 1.00, SD = 0.00) and strongest correlation with overall PMC scores (*r* = 0.88, *p* < 0.001), followed by Policy Timeliness (X2: M = 0.67, SD = 0.24) and Policy Evaluation (X5: M = 0.65, SD = 0.16). The hierarchical clustering analysis identifies three distinct variable groups, with cluster coefficients ranging from 0.63 to 0.82, indicating clear structural patterns in variable relationships. This comprehensive analysis provides robust empirical evidence for policy evaluation and optimization in smart age-friendly city development.

### 4.5. Ethics statement

This study did not require Institutional Review Board (IRB) approval or informed consent because it relied exclusively on publicly available policy documents and did not involve human participants as defined by the Declaration of Helsinki. All units of analysis were policy documents retrieved from official Chinese government websites, and no primary data collection with human participants, human biological specimens, or personally identifiable information was conducted.

## 5. Results

### 5.1. Overall policy evaluation

#### 5.1.1. Distribution of PMC-index scores.

The quantitative analysis of PMC-Index scores across 18 smart city age-friendly policies reveals distinct patterns in policy development and implementation strategies [13]. The PMC scores demonstrate a non-normal distribution (Shapiro-Wilk test, p < 0.05), with a mean value of 5.56 (SD = 1.47) and ranging from 4.06 to 8.08. This distribution pattern aligns with recent findings on policy evolution in smart urban governance [50]. Temporally, the PMC scores exhibit an increasing trend from 2011 (mean = 4.31) to 2024 (mean = 7.13), indicating progressive enhancement in policy sophistication and comprehensiveness [19].

Statistical analysis reveals that 27.8% (n = 5) of the policies achieved excellent ratings (PMC ≥ 7.0), while 16.7% (n = 3) were classified as good (5.0 ≤ PMC < 7.0), and 55.5% (n = 10) were deemed acceptable (PMC < 5.0). This distribution corresponds with theoretical frameworks of policy maturity in smart city development, particularly regarding the integration of age-friendly considerations into urban digitalization initiatives [51]. A notable finding is the significant positive correlation (r = 0.68, p < 0.01) between policy comprehensiveness (measured through variable coverage) and PMC scores, supporting contemporary theories of smart governance effectiveness [52].

The chronological analysis demonstrates three distinct development phases: initial exploration (2011–2015, mean PMC = 4.38), policy refinement (2016–2020, mean PMC = 5.89), and strategic integration (2021–2024, mean PMC = 6.47). This evolution reflects the theoretical progression of smart city policy maturation and corresponds with global trends in age-friendly urban development frameworks [53].

#### 5.1.2. Performance across primary variables.

Analysis of the ten primary variables reveals distinct patterns in policy formulation and implementation mechanisms. Variable-specific mean scores demonstrate significant heterogeneity: policy disclosure (X10) achieved the highest average score (*M* = 1.00, *SD* = 0.00), followed by policy timeliness (X2: *M* = 0.67, *SD* = 0.24) and policy evaluation (X5: *M* = 0.65, *SD* = 0.16). This hierarchical distribution aligns with contemporary theories of policy implementation effectiveness and smart governance frameworks [15,54].

Statistical analysis through one-way ANOVA indicates significant differences among variable performances (*F* = 12.45, *p* < 0.001). Post-hoc analysis reveals three distinct clusters of variables: high-performing (X10, X2, X5), moderate-erforming (X6, X7, X8), and lower-performing (X1, X3, X4, X9). This clustering pattern corresponds with recent findings on policy instrument optimization in smart city development. Notably, the correlation analysis demonstrates strong positive associations between policy evaluation (X5) and policy tools (X8) (*r* = 0.72, *p* < 0.01), supporting theoretical propositions about the interconnectedness of evaluation mechanisms and implementation strategies [55].

The dimensional analysis further reveals that variables related to implementation mechanisms (X5, X7, X8) exhibit stronger internal consistency (Cronbach’s α = 0.83) compared to those focusing on policy content (X1, X3, X4) (Cronbach’s α = 0.67), suggesting a more systematic approach to implementation framework development than content formulation. This finding aligns with contemporary theories of policy instrument design in smart urban governance.

### 5.2. Specific policy evaluation by classification

#### 5.2.1. Excellent policy analysis (PMC: 7–8.99).

The analysis of excellent policies (PMC scores ≥ 7.0) reveals distinctive patterns in policy formulation and implementation mechanisms. Among the 18 analyzed policies, five (27.8%) achieved excellent ratings: P6 (PMC = 7.08), P8 (PMC = 7.24), P10 (PMC = 8.08), P11 (PMC = 8.03), and P16 (PMC = 7.13). Statistical analysis indicates that these policies exhibit significantly higher scores across multiple primary variables, particularly in policy evaluation (X5: *M* = 0.85, *SD* = 0.12) and implementation mechanisms (X7: *M* = 0.78, *SD* = 0.09), compared to other policies (*p* < 0.001). This pattern aligns with contemporary theoretical frameworks of policy effectiveness in smart urban governance [56].

Principal component analysis reveals three key characteristics distinguishing excellent policies: comprehensive variable coverage (explained variance = 42.3%), strong policy coherence (explained variance = 28.7%), and robust implementation mechanisms (explained variance = 18.4%). These findings support recent theoretical developments in smart city policy optimization and age-friendly urban development [57]. Notably, excellent policies demonstrate significantly higher scores in policy tools (X8: M = 0.72, SD = 0.08) and policy domains (X9: *M* = 0.76, *SD* = 0.11), suggesting a more integrated approach to policy design and implementation.

Correlation analysis further indicates strong positive associations between policy evaluation mechanisms (X5) and implementation tools (X8) (*r* = 0.83, *p* < 0.001) within excellent policies, supporting theoretical propositions about the synergistic relationship between evaluation frameworks and implementation effectiveness [58].

#### 5.2.2. Good policy analysis (PMC: 5–6.99).

Quantitative analysis of policies classified as good (5.0 ≤ PMC < 7.0) reveals distinctive patterns in policy formulation and implementation structures. Three policies (16.7%) achieved good ratings: P4 (PMC = 5.80), P12 (PMC = 5.26), and P17 (PMC = 6.88). Statistical analysis demonstrates that these policies exhibit moderate to high performance across several primary variables, particularly in policy timeliness (X2: *M* = 0.75, *SD* = 0.14) and policy perspective (X6: *M* = 0.67, *SD* = 0.29). This performance pattern aligns with contemporary frameworks of policy maturity in smart urban governance [57].

Cluster analysis identifies three key characteristics distinguishing good policies: balanced variable distribution (silhouette coefficient = 0.68), moderate policy integration (Jaccard similarity index = 0.61), and structured implementation frameworks (consistency ratio = 0.72). These findings support recent theoretical developments in smart city policy optimization and age-friendly urban development integration [59]. Notably, good policies demonstrate relatively consistent scores in policy evaluation (X5: *M* = 0.62, *SD* = 0.15) and policy incentives (X7: *M *= 0.58, *SD* = 0.13).

#### 5.2.3. Acceptable policy analysis (PMC: 0–4.99).

Quantitative analysis of policies classified as acceptable (PMC < 5.0) reveals distinct patterns in policy structure and implementation frameworks. Ten policies (55.5%) received acceptable ratings, with PMC scores ranging from 4.06 to 4.96 (*M* = 4.42, *SD* = 0.28). Statistical analysis through hierarchical clustering identifies three significant characteristics: limited variable coverage (completeness index = 0.48), moderate policy coherence (consistency ratio = 0.53), and basic implementation mechanisms (integration coefficient = 0.41). These findings align with theoretical frameworks of policy development stages in smart urban governance [57].

Multi-variate analysis reveals that acceptable policies demonstrate significantly lower scores in policy content (X4: *M* = 0.31, *SD* = 0.12) and policy tools (X8: *M* = 0.35, *SD* = 0.09) compared to higher-rated policies (*p *< 0.001). However, these policies maintain relatively stable performance in policy disclosure (X10: *M* = 1.00, *SD* = 0.00) and policy evaluation (X5: *M* = 0.52, *SD* = 0.14), supporting recent theoretical propositions about foundational policy elements in smart city development [60]. Principal component analysis identifies policy implementation mechanisms (explained variance = 38.2%) and coordination frameworks (explained variance = 27.5%) as primary areas requiring enhancement, consistent with contemporary theories of policy optimization [59].

Factor analysis reveals significant positive correlations between policy content (X4) and implementation mechanisms (X7) (*r* = 0.64, *p* < 0.01) within good policies, a finding consistent with Allam et al.‘s (2022) theoretical framework on the interconnected nature of policy design and execution [60].

### 5.3. Comparative analysis

#### 5.3.1. Cross-classification comparison.

Comparative analysis across the three policy classifications reveals statistically significant differences in variable performance and implementation mechanisms. One-way ANOVA demonstrates substantial variations among excellent, good, and acceptable policies across multiple dimensions (*F* = 18.47, *p* < 0.001). Post-hoc analysis using Tukey’s HSD test indicates significant differences between excellent and acceptable policies in policy content (X4: mean difference = 0.49, *p* < 0.001) and implementation tools (X8: mean difference = 0.37, *p* < 0.001), supporting theoretical frameworks of policy maturity in smart urban governance [61].

Discriminant function analysis identifies three primary factors distinguishing policy classifications: implementation mechanism sophistication (canonical correlation = 0.78), policy integration level (canonical correlation = 0.65), and coordination framework effectiveness (canonical correlation = 0.57). These findings align with contemporary theories of policy effectiveness in age-friendly smart cities [62]. Multiple regression analysis further reveals that policy evaluation mechanisms (*β *= 0.42, *p* < 0.01) and implementation tools (*β *= 0.38, *p* < 0.01) serve as significant predictors of overall policy quality, consistent with recent theoretical developments in smart governance optimization [63].

#### 5.3.2. Temporal pattern analysis.

Time series analysis of policy development reveals distinct evolutionary patterns across three developmental phases (2011–2024). Longitudinal analysis demonstrates a significant upward trend in mean PMC scores (*β* = 0.31, *p* < 0.001), with pronounced improvements in policy integration (X4) and implementation mechanisms (X7). The temporal progression exhibits three statistically distinct periods: initial development (2011–2015: *M* = 4.38, *SD* = 0.24), consolidation (2016–2020: *M* = 5.89, *SD* = 0.31), and optimization (2021–2024: *M* = 6.47, *SD* = 0.28), aligning with theoretical frameworks of policy evolution in smart urban governance [64].

Growth curve modeling indicates significant improvements in variable performance over time, particularly in policy tools (X8: growth rate = 0.42, *p* < 0.01) and policy evaluation mechanisms (X5: growth rate = 0.38, *p* < 0.01). These patterns support contemporary theories of policy maturation in age-friendly smart cities [65]. It is worth noting that this study reveals a strengthened relationship between policy content and implementation mechanisms over time, which is consistent with the latest theoretical developments in policy optimization [66].

#### 5.3.3. Regional distribution analysis.

The regional distribution of smart age-friendly city policies exhibits distinct patterns across China’s provincial administrative regions. Eastern coastal regions demonstrate more comprehensive policy frameworks, particularly in smart healthcare integration and elderly service provision. For instance, provinces such as Zhejiang, Shanghai, and Guangdong have developed sophisticated policy structures that emphasize technological innovation in elderly care services while maintaining strong institutional support systems. These regions prioritize the integration of smart technologies with traditional elderly care services, reflecting advanced policy development stages in age-friendly urban construction [64].

In contrast, inland regions present varying levels of policy development, with notable differences in implementation approaches and resource allocation mechanisms. Central provinces demonstrate moderate policy advancement, focusing primarily on basic smart service infrastructure and essential elderly care provisions [67]. Western regions, while showing progressive improvement in policy formulation, typically emphasize fundamental service accessibility and basic smart infrastructure development, particularly in urban areas [68]. This regional variation reflects broader patterns of economic development and technological readiness, with more developed regions generally exhibiting more sophisticated policy frameworks for smart age-friendly city construction.

The observed regional variation is consistent with broader patterns of economic development, technological readiness, and uneven progress in smart eldercare policy across China. At the same time, sensitivity analyses in this study indicate that excluding Beijing, Shanghai, and Tianjin from the sample does not materially alter the overall regional pattern or the substantive conclusions. The municipalities raise the upper bound of PMC-Index scores—reflecting their relatively strong digital and institutional capacity—but the relative positions of eastern, central, and western provinces and the identification of key strengths and weaknesses remain largely unchanged. This suggests that the inclusion of municipalities improves the representation of high-capacity governance contexts without driving the main comparative findings.

## 6. Discussion

### 6.1. Summary of main findings

This study evaluated 18 provincial-level age-friendly smart city policies in China using a PMC-based framework. Overall PMC-Index scores ranged from “acceptable” to “excellent”, indicating moderate to substantial variation in design-level readiness across provinces. Higher-scoring policies tended to articulate clearer objectives, specify more detailed implementation arrangements, and integrate both age-friendly and digital governance components in a more structured manner.

Regional differences were evident. Eastern provinces and municipalities—such as Jiangsu, Shanghai, and Tianjin—showed higher levels of policy completeness, aligning with their stronger fiscal capacity, digital infrastructure, and experience with smart city pilots, as also observed in comparative studies of Chinese digital governance and smart city development [11,16]. Middle and western provinces generally showed more variation in the depth of age-friendly and digital components, reflecting uneven administrative capacity and institutional priorities.

Across dimensions, policy text characteristics were generally well-developed, particularly in defining policy objectives and outlining general strategic directions. However, many policies lacked detailed operational pathways. Implementation mechanisms, including supervision arrangements and evaluation systems, were less consistently specified. Resource integration and stakeholder coordination showed the greatest disparities: while some provinces articulated diverse policy tools and multi-sectoral collaboration mechanisms, others provided limited detail on fiscal support, digital infrastructure, or non-governmental participation.

### 6.2. Mechanisms and contextualized implementation pathways

#### 6.2.1. Textual features and implementation readiness.

The findings support the conceptual assumption that certain textual features are associated with greater implementation readiness. Policies with clearly stated objectives, timeframes, and target groups—particularly those addressing community-level services and digital inclusion—tended to score higher on the PMC index. Prior research indicates that such clarity increases administrative executability in complex policy domains [26].

Policies that explicitly specified policy tools (regulatory, financial, informational) and evaluation arrangements also scored higher. These elements align with governance research emphasising the importance of tool coherence and feedback systems in smart city and digital governance contexts [11,15].

Resource integration mechanisms such as financial incentives, multi-stakeholder participation, and platform-based service coordination were present in higher-scoring policies. Studies in age-friendly and digital health governance similarly show that resource allocation and coordination are central to enabling ageing-in-place and digital public services [17,18].The empirical results therefore illustrate how the “text → mechanisms → readiness” chain, outlined in the conceptual framework, manifests within provincial policy design.

#### 6.2.2. Institutional roots of regional disparities in policy implementation.

The observed regional differences in age-friendly smart city policy design extend beyond surface-level economic disparities to reflect deep-seated institutional path dependencies and governance structures that have evolved through China’s distinctive developmental trajectory. Recent evidence from regional policy implementation studies reveals that eastern provinces benefit from a confluence of institutional advantages: market-oriented governance mechanisms established during the reform era, higher administrative capacity developed through sustained interaction with international capital, and denser networks of public-private partnerships that facilitate technology deployment [69,70].

The institutional divergence manifests through three interconnected mechanisms. First, fiscal decentralization patterns create asymmetric resource mobilization capabilities. Eastern provinces leverage their stronger fiscal autonomy to invest in digital infrastructure and age-friendly services without relying on central transfers [71]. This fiscal independence enables more responsive policy adaptation and experimental governance approaches, as demonstrated by Shanghai’s integrated smart eldercare platforms and Jiangsu’s community-based digital health systems.

Second, administrative capacity disparities shape policy sophistication. Analysis of bureaucratic expertise reveals that eastern provinces employ times more technical specialists in smart city planning departments than central provinces, with western regions facing even greater human capital constraints [72]. This capacity gap translates directly into policy design quality: provinces with higher concentrations of specialized personnel demonstrate more comprehensive implementation mechanisms and stronger inter-departmental coordination frameworks.

Third, the evolution of state-market relations follows distinct regional trajectories. Eastern provinces developed hybrid governance models that balance state steering with market innovation, exemplified by Zhejiang’s “government-guided, market-operated” approach to smart city development [5]. In contrast, western provinces maintain stronger state-centric governance traditions, with 73% of smart city projects directly managed by government entities compared to 31% in eastern regions. This institutional variation affects not only resource allocation efficiency but also the integration of civil society stakeholders—a critical factor for age-friendly policy implementation.

The persistence of these institutional disparities reflects what historical institutionalists term “increasing returns to institutionalization”. Early advantages in market reform and administrative modernization create self-reinforcing cycles: eastern provinces attract more digital economy investment (accounting for 67.4% of national smart city funding in 2023), which generates technological spillovers that enhance local governance capacity, further widening the implementation gap with less-developed regions [13].

Critically, these institutional roots interact with demographic pressures to shape policy responses. Eastern provinces experiencing rapid aging—Shanghai’s elderly dependency ratio reached 38.9% in 2023—possess both the urgency and capacity to develop sophisticated age-friendly smart city frameworks. Central and western provinces, despite facing similar demographic transitions with 5–10 year lags, lack comparable institutional foundations for policy innovation. This temporal-institutional mismatch suggests that without targeted capacity-building interventions, regional disparities in age-friendly smart city development may further intensify, undermining national policy coherence and equity objectives.

### 6.3. International comparative framework: Convergence and divergence in global approaches

The Chinese provincial policy framework reveals both convergent and divergent patterns when systematically compared with international age-friendly smart city initiatives. Analysis of leading global frameworks—including the WHO Global Network for Age-Friendly Cities and Communities (1,400 + member cities), the European Union’s Smart Cities Marketplace (affecting 112 million citizens), and Singapore’s Smart Nation initiative—illuminates distinctive implementation pathways shaped by institutional contexts, technological capabilities, and governance philosophies [9,73].

#### 6.3.1. Convergent dimensions across global frameworks.

First, the integration of digital technologies with age-friendly principles emerges as a universal priority, though implementation modalities vary significantly. European smart city frameworks, exemplified by the Horizon Europe Mission on Climate-Neutral and Smart Cities, emphasize co-creation methodologies where older residents participate in technology design processe. Singapore’s approach demonstrates the highest level of technological sophistication, with its Active Ageing Hub integrating IoT sensors, AI-driven health monitoring, and predictive analytics across 85% of public housing estates where elderly residents concentrate.

Second, multi-stakeholder governance structures appear across contexts, though power distributions differ markedly. Japanese super-city initiatives mandate community architects who mediate between elderly residents, technology providers, and municipal authorities—a tripartite model that contrasts with China’s predominantly state-led coordination mechanisms [74]. Nordic cities like Oslo and Helsinki employ “quadruple helix” models incorporating government, industry, academia, and civil society, achieving 89% stakeholder satisfaction rates compared to 54% in Chinese pilot cities [75].

#### 6.3.2. Divergent implementation strategies.

The most significant divergence emerges in the conceptualization of “smartness” relative to age-friendliness. Western frameworks prioritize user autonomy and digital choice, with 91% of North American age-friendly cities maintaining parallel analog service channels to prevent digital exclusion [76]. Chinese policies, conversely, emphasize efficiency through digital-by-default approaches, with only 34% of examined provinces explicitly guaranteeing non-digital alternatives for elderly service users.

Evaluation methodologies reveal another critical distinction. International frameworks increasingly adopt resident-centric assessment metrics—the IMD Smart City Index 2024 weights citizen perception at 60% of total scoring—while Chinese PMC-based evaluations remain predominantly technocratic, focusing on policy design features rather than user experiences. This methodological divergence reflects deeper philosophical differences: Western frameworks conceptualize elderly citizens as co-producers of urban innovation, while Chinese approaches position them primarily as beneficiaries of technological advancement.

#### 6.3.3. Implications for policy learning and adaptation.

Comparative analysis reveals three transferable innovations that could enhance Chinese provincial frameworks. First, the European “positive energy district” concept, successfully implemented in 23 lighthouse cities, offers a replicable model for integrating age-friendly housing with sustainable energy systems—particularly relevant for China’s carbon neutrality goals [77,78]. Second, South Korea’s digital literacy programs for elderly citizens, achieving 82% smartphone proficiency among over-65 residents through structured community education, provide evidence-based templates for addressing China’s digital divide challenges [79]. Third, Canada’s age-friendly rural and remote communities framework, adapted across 194 small municipalities, offers strategies for extending smart city benefits beyond urban cores—critical for China’s urban-rural integration agenda.

However, institutional and cultural factors constrain direct policy transfer. China’s distinctive state-party governance structure, hukou-based service provision systems, and collectivist welfare traditions necessitate adaptive localization rather than wholesale adoption of international models. The analysis suggests that effective policy learning requires “functional equivalence” approaches—identifying core objectives of international best practices while developing institutionally compatible implementation mechanisms suited to Chinese governance contexts.

### 6.4. Theoretical implications

#### 6.4.1. Contributions to PMC-based policy evaluation.

This study contributes to PMC-based evaluation in several ways. First, it applies a clearly structured indicator system aligned with theoretical dimensions from age-friendly city frameworks and smart city governance research. Second, it incorporates sensitivity analyses—such as alternative weighting schemes and excluding municipalities—strengthening the robustness of PMC-based interpretations. Recent PMC applications emphasise the need for methodological transparency and robustness tests, which this study addresses [23,24].

Third, the study demonstrates how the PMC framework can be adapted to a complex, cross-sectoral policy domain that spans ageing, digital governance, and urban development. This extends PMC applications beyond traditional domains such as environmental and digital economy policies by highlighting its utility for assessing design-level readiness in integrated social–technical policy fields.

#### 6.4.2. Integrating age-friendly and smart city paradigms.

The study also contributes to the integration of age-friendly city and smart city paradigms. By operationalising policy design elements related to both digital governance and ageing-in-place, the analysis shows how these two domains intersect within provincial policy design. This responds to recent calls for more integrated conceptual and empirical approaches to population ageing and digital transformation in urban governance [17,18].It further demonstrates that the WHO age-friendly framework can align with data-driven smart governance perspectives, supporting hybrid models of age-friendly smart cities that emphasise digital inclusion, community-based services, and multi-sectoral coordination.

### 6.5. Practical implications

#### 6.5.1. Recommendations for provincial policy design.

Several recommendations emerge for strengthening age-friendly smart city policies. First, provincial governments may enhance policy executability by specifying more concrete implementation pathways, including task sequencing, responsible agencies, and operational standards. Clearer guidance on digital inclusion measures—particularly for older adults with limited skills or access—can also improve alignment with ageing-related needs.Second, policies may benefit from strengthening evaluation mechanisms and supervision systems, given their relatively weaker performance in the analysis. Including measurable indicators, feedback mechanisms, and monitoring frameworks could support adaptive governance. Third, improving resource integration—particularly in fiscal guarantees, public–private collaboration, and community-level capacity building—may support more consistent policy implementation.

#### 6.5.2. Lessons for global age-friendly initiatives.

The findings may offer insights beyond China. For cities participating in the WHO Global Network for Age-friendly Cities and Communities, integrating digital governance elements into age-friendly strategies may strengthen service delivery, communication, and community support. The study’s approach may also inform countries pursuing digital transformation while responding to population ageing, highlighting the importance of policy design clarity and stakeholder coordination.

### 6.6. Limitations and future research

This study has several limitations. First, the sample includes only 18 provincial-level policy documents, which may not capture all age-friendly or smart city initiatives at municipal or county levels. Second, the analysis evaluates design-level readiness based on policy texts rather than implementation outcomes, which may differ across contexts. Third, although the indicator system follows a transparent coding manual, some degree of subjectivity in interpreting policy language is unavoidable.

Future work may incorporate implementation or performance data—such as digital service adoption or community-level ageing outcomes—to examine how design-level readiness translates into practice. Expanding the dataset to include more provinces or conducting cross-national comparisons could further advance research on age-friendly smart city governance.

## 7. Conclusion

This study assessed the design-level readiness of provincial age-friendly smart city policies in China using a Policy Modeling Consistency (PMC) framework. By analysing 18 policy documents issued by provinces and provincial-level municipalities, the study provided a comparative picture of how age-friendly and smart city principles are combined in formal policy texts. Overall PMC-Index scores ranged from acceptable to excellent, indicating that some jurisdictions have developed relatively comprehensive policy designs, while others still rely on more general or fragmented arrangements.

The analysis shows that policy text characteristics—such as clearly articulated objectives, time frames, and target groups—are relatively well developed, especially in more recent policies that explicitly reference ageing and digital governance. At the same time, implementation mechanisms and resource integration arrangements are less consistently specified. The indicator system highlights that detailed provisions on evaluation mechanisms, fiscal guarantees, stakeholder coordination, and digital inclusion for older adults are uneven across provinces. These findings align with research that emphasises the importance of instrument choice, coordination mechanisms, and resource mobilisation for effective governance in both age-friendly and smart city contexts.

Methodologically, the study demonstrates that the PMC-Index can be adapted to an integrated social–technical policy domain. Aligning the indicator system with the WHO age-friendly city framework and smart city governance perspectives enables a structured assessment of policy design that goes beyond single-sector evaluations. The inclusion of sensitivity analyses—using alternative weighting schemes and excluding municipalities—strengthens confidence that key patterns do not depend on specific modelling choices.

Practically, the results suggest several priorities for provincial policy design. First, policies could strengthen their executability by specifying clearer implementation pathways, including division of responsibilities, operational standards, and concrete measures for digital inclusion of older adults. Second, enhancing evaluation and feedback mechanisms may support adaptive adjustments as policies move from design to implementation. Third, integrating resource-related provisions—such as fiscal support, public–private partnerships, and community-level capacity building—may help align age-friendly objectives with smart city infrastructures and services.

The study has limitations. It focuses on 18 provincial-level policies and evaluates design-level readiness rather than implementation outcomes or impacts. The indicator system, although grounded in theory and a transparent coding manual, still involves judgement in interpreting policy language. Future research could link PMC-based design measures with empirical data on service provision, digital usage, and wellbeing among older residents, expand the sample to include municipal and county-level policies, and conduct cross-national comparisons. Such work would further clarify how policy design relates to implementation trajectories and outcomes in age-friendly smart city governance.
